# Single-Cell FISH Analysis Reveals Distinct Shifts in PKM Isoform Populations during Drug Resistance Acquisition

**DOI:** 10.3390/biom12081082

**Published:** 2022-08-06

**Authors:** Seong Ho Kim, Ji Hun Wi, HyeRan Gwak, Eun Gyeong Yang, So Yeon Kim

**Affiliations:** 1 Chemical and Biological Integrative Research Center, Biomedical Research Division, Korea Institute of Science and Technology, Seoul 02792, Korea; 2 Department of Biotechnology, College of Life Sciences and Biotechnology, Korea University, Seoul 02792, Korea; 3 Division of Bio-Medical Science and Technology, Korea University of Science and Technology (UST), KIST Campus, Seoul 02792, Korea

**Keywords:** drug response, isoform regulation, pyruvate kinase m (pkm), single-cell analysis

## Abstract

The Warburg effect, i.e., the utilization of glycolysis under aerobic conditions, is recognized as a survival advantage of cancer cells. However, how the glycolytic activity is affected during drug resistance acquisition has not been explored at single-cell resolution. Because the relative ratio of the splicing isoform of pyruvate kinase M (PKM), PKM2/PKM1, can be used to estimate glycolytic activity, we utilized a single-molecule fluorescence in situ hybridization (SM-FISH) method to simultaneously quantify the mRNA levels of PKM1 and PKM2. Treatment of HCT116 cells with gefitinib (GE) resulted in two distinct populations of cells. However, as cells developed GE resistance, the GE-sensitive population with reduced PKM2 expression disappeared, and GE-resistant cells (Res) demonstrated enhanced PKM1 expression and a tightly regulated PKM2/PKM1 ratio. Our data suggest that maintaining an appropriate PKM2 level is important for cell survival upon GE treatment, whereas increased PKM1 expression becomes crucial in GE Res. This approach demonstrates the importance of single-cell-based analysis for our understanding of cancer cell metabolic responses to drugs, which could aid in the design of treatment strategies for drug-resistant cancers.

## 1. Introduction

Recent advances in various cancer therapies, including chemotherapeutic drugs, targeted anticancer drugs, and cancer immunotherapy, have extended the lifespan of cancer patients. However, the acquisition of resistance remains a major obstacle for all the above-mentioned cancer therapeutics. As cancer cells are heterogeneous, even within tissues derived from a single patient, the entire process of development of drug resistance in cancer cells should be considered at the single-cell level. Several proposed drug resistance mechanisms originate from cancer cell evolution, heterogeneity, and the tumor microenvironment [1,2], the effects of which can be assessed by tracking and observing individual cells [3].

The Warburg effect describes the utilization of glycolysis over oxidative phosphorylation, even under aerobic conditions, and has been recognized as a survival advantage of tumor cells. Specifically, the Warburg effect promotes epigenetic and genetic changes, leading to enhanced cellular heterogeneity [4]. Cancer stem cells (CSCs) usually exhibit enhanced glycolysis due to their localization within hypoxic tumor regions, and genetic changes in CSCs support their survival by enhancing drug treatment resistance. Importantly, drug-resistant cancer cells usually exhibit an increased rate of glycolysis, suggesting that understanding the metabolic status of individual cells may lead to therapeutic strategies to reduce drug resistance (see [4,5,6] for review). A recent study involved the analysis of thousands of metabolic genes to elucidate metabolic heterogeneity at a single-cell resolution, thereby illustrating that cancer cells are more “flexible” and exhibit upregulated metabolic activity [7]. However, the link between cancer metabolic activity and drug resistance at a single-cell level has not been explored.

Among the various enzymes involved in glycolysis, pyruvate kinase M (PKM) is a rate-limiting glycolytic enzyme that catalyzes the conversion of phosphoenolpyruvate (PEP) and adenosine diphosphate (ADP) to pyruvate and adenosine triphosphate (ATP) [8]. Specifically, splice isoforms PKM1 and PKM2 have been extensively studied due to their critical roles in glycolysis and signaling. The PKM1 tetramer exhibits higher pyruvate kinase activity compared to the less active PKM2 tetramer, which induces a metabolic shift toward enhanced glycolysis. Additionally, PKM2 can dissociate into dimers and function as a protein kinase, transcriptional regulator, and extracellular communicator. All these functions are beneficial in terms of promoting the Warburg effect and tumor growth [9,10]. Therefore, it has been suggested that in cancer cells, the predominant isoform shifts from PKM1 to PKM2, which enhances glycolysis, drug resistance, and tumorigenesis [11]. Extensive studies have been conducted to elucidate the effect of PKM2 on drug sensitivity and resistance [12,13,14,15,16,17,18,19]. In addition to these studies of PKM2, a relationship between PKM1 and drug resistance has also been identified [20,21]. However, these results are controversial, as the expression levels of PKM1 and PKM2, as well as their relative expression (PKM2/PKM1 ratio), vary significantly depending on the cell and tissue type [22,23,24], whereas most of the studies discussed above focused on one specific isoform, thereby ignoring the effects of the other isoform. The detailed mechanisms correlating PKM isoforms and resistance can be elucidated by examination of cells during the process of acquiring drug resistance, as opposed to a simple comparison of two conditions (e.g., untreated cells and drug-treated cells or untreated cells and drug-resistant cells). As cancer cells are heterogeneous and drugs usually kill a fraction of cells, it is critical to understand the role of splicing variability at the single-cell level and track which population of cells survives. Such insights could bring us closer to a complete understanding of the role of glycolytic enzymes in the acquisition of drug resistance [3].

Recently developed imaging techniques have allowed for observation of single RNA molecules in cells, tissues, and embryos. Owing to its high spatiotemporal sensitivity and the ability to precisely quantify multiple endogenous mRNAs at the same time, single-molecule RNA imaging has become a powerful technique for RNA sequencing at the single-cell level [25]. Endogenous RNA molecules and their splicing variants can be detected and distinguished by single-molecule fluorescence in situ hybridization (SM-FISH) [26]. Additionally, single-cell RNA imaging has been utilized to elucidate how certain cell populations acquire drug resistance [27]. Because the relative ratio of PKM1 and PKM2 can be used to estimate metabolic status and glycolytic activity [11,28], we carefully designed an SM-FISH study to quantify the expression of these two PKM isoforms with a 20-base-pair difference at a single-cell level. We utilized this method to study the role of metabolic shift in the acquisition of drug resistance. Notably, upon drug treatment, we were able to identify two subpopulations based on PKM2 expression that could not be distinguished by conventional measurements. Additionally, drug-resistant cells showed enhanced PKM1 expression with a tightly regulated ratio of PKM2 to PKM1. These observations suggest that each PKM isoform has a distinct function in drug response and resistance in colorectal cancer cells.

## 2. Materials and Methods

### 2.1. Materials

Fetal bovine serum (FBS) was obtained from Life Technologies (Waltham, MA, USA). RPMI and MEM medium were purchased from Welgene Inc. (Gyeongsan, Korea). The antibodies against PKM1 (21577) and PKM2 (21578) were obtained from Signalway (Greenbelt, MD, USA). Lamin B1 antibody (12586), horseradish peroxidase (HRP)-conjugated goat anti-mouse (7076) and anti-rabbit secondary antibodies (7074) were obtained from Cell Signaling Technology (Danvers, MA, USA). β-actin antibody (A2228), vinculin antibody (V9131), and all the chemicals, including DMSO (D8418), gefitinib (GE, SML1657), dextran sulfate (D8906), vanadyl-ribonucleoside complex (94742), *E. coli* RNAse-free tRNA (10109541001), and formamide (221198), were purchased from Sigma-Aldrich (St. Louis, MO, USA). The L-Lactate assay kit (ab65330) and the extracellular oxygen consumption assay (ab197243) were purchased from Abcam (Cambridge, UK). 3-(4,5-dimethylthiazol-2-yl)-2,5-diphenyltetrazolium bromide (MTT) was purchased from Invitrogen (Waltham, MA, USA).

### 2.2. Cell Culture

Human colorectal cancer cell lines, HCT116 cells, HCT15 cells, and Caco-2 cells (Korean Cell Line Bank, Seoul, Korea) were grown in RPIM (HCT15 and HCT116) and MEM (Caco-2) medium supplemented with 10% fetal bovine serum and 1% penicillin/streptomycin at 37 °C in a 5% CO_2_ atmosphere. To prepare GE-resistant cells, 1.5 × 10^5^ of HCT116 cells were initially cultured in the presence of 10 μM of GE for 24 h. The surviving cells were regrown in the drug-free medium until at least 1.2 × 10^5^ cells were obtained. Cells were repeatedly cultured by a stepwise increase in GE concentration up to 150 μM. Under these conditions, we confirmed that EGFR phosphorylation was not affected by GE treatment. After checking whether HCT116 obtained GE resistance, cells were maintained in the presence of 20 μM of GE.

### 2.3. Semi-Quantitative Reverse Transcription (RT)-PCR and Real-Time Quantitative PCR (qRT-PCR)

First, 1 × 10^5^ of HCT116 cells seeded in a 6-well plate were treated with either DMSO or GE and incubated for 24 h. Total RNA was purified using a GeneJET RNA purification kit (Thermo Scientific). From the purified RNA, cDNA was synthesized using a TOP scriptTM cDNA synthesis kit (Enzynomics, Daejeon, Korea). All of the primers used in this study were obtained from Macrogen (Seoul, Korea) and are listed in Table S2. The detailed protocol for PCR included 28 cycles of denaturation at 94 °C for 45 s, annealing at 57 °C for 45 s, and elongation at 72 °C for 45 s. A volume of 10 μL of the PCR product was electrophoresed on a 1.5% agarose gel and visualized using a MiniBIS Pro gel documentation system (DNR Bioimaging Systems, Neve Yamin, Israel). For RT-qPCR, cDNA was amplified using Power SYBR Green PCR Master Mix (Thermo Scientific) on a QuanStudio 1 real-time PCR instrument (Applied Biosystems, Waltham, MA, USA). Relative mRNA levels were normalized to GAPDH mRNA levels. The relative expression levels were determined by comparative Ct method. Primer sequences are listed in Table S2.

### 2.4. Cell Viability Measurement

To measure cell viability by trypan blue staining, 1.5 × 10^5^ cells were seeded in 6-well plates. After 24 h, cells were treated with appropriate reagents, as described in the main text. Then, cells were detached and mixed with 0.4% trypan blue (Sigma Aldrich, St. Louis, MO, USA) to exclude dead cells, and viable cells were selectively counted.

### 2.5. RNA Interference

Scrambled siRNAs for control measurements, as well as PKM1 and PKM2 siRNAs, were purchased from Bionner Inc. (Daejeon, Korea). The following siRNAs were used: scrambled siRNA (no. SN-1003), PKM1-directed siRNA (sense strand: GCGTGGAGGCTTCTTATAA, antisense stand: CGCACCTCCGAAGAATATT) [29], and PKM2-directed siRNA (sense strand: CCATAATCGTCCTCACCAA, antisense stand: GGTATTAGCAGGAGTGGTT) [29]. The siRNAs were introduced into cells in Opti-MEM (GIBCO, USA) using Lipofectamine RNAiMAX reagent (Invitrogen, Waltham, MA, USA) as instructed. Scrambled siRNAs were utilized for all of the control measurements.

### 2.6. Preparation of FISH Probe for Hybridization

All SM-FISH probes were purchased from Integrated DNA Technologies (Newark, NJ, USA) and are listed in Table S1. First, hybridization buffer containing 10% dextran sulfate, 2 mM vanadyl-ribonucleoside complex, 40 μg/mL of *E. coli* RNAse-free tRNA in 2X SSC (300 mM NaCl, 30 mM sodium citrate (pH 7.0), and 0.04% SDS was prepared. Varying amounts (50%, 30%, and 20%) of formamide were added for hybridization of primary, secondary, and tertiary probes, respectively. For the hybridization reaction, 40 μL of 50 nM probe dissolved in hybridization buffer was prewarmed for 5 min at 37 °C prior to use.

### 2.7. In Situ Hybridization

Cells on a confocal dish were washed with 1X PBS (100 mM Na2HPO4, 20 mM KH2PO4, 137 mM NaCl, 27 mM KCl, pH 7.4) and fixed for 10 min at room temperature in 1X PBS with 3.6% formaldehyde and 10% acetic acid. After fixation, cells were washed twice with 1X PBS and permeabilized by 70% ethanol overnight at 4 °C. Cells were prewashed with wash buffer containing 50% formamide, 2X SSC, and 0.04% SDS. Then, primary hybridization was achieved with 40 μL of primary hybridization solution overnight at 37 °C. During the hybridization processes, the confocal dish was placed into a large container with wet paper towel to prevent evaporation of the buffer. A coverslip was used to spread the hybridization solution over the entire surface. After primary hybridization, cells were washed three times with wash buffer (2X SSC, 0.04% SDS, and 30% formamide) for 10 min. Then, secondary probe was added and incubated for 3 h at 37 °C. Following secondary hybridization, cells were washed three times with wash buffer (2X SSC, 0.04% SDS, and 20% formamide) for 10 min. Lastly, tertiary probe reactions with cells on a confocal dish were performed by placing them over a drop of tertiary hybridization. The samples were covered with aluminum foil and incubated for 3 h at 37 °C. Thereafter, cells were washed three times with wash buffer (2X SSC, 0.04% SDS, and 20% formamide) for 10 min. The buffer was changed to 2X SSC for imaging.

### 2.8. SM-FISH Imaging and Analysis

Single-molecule RNA images were acquired with a homemade setup constructed with an Olympus IX 73 inverted microscope as described previously [30]. Briefly, AX488- and Cy3-labeled probes were excited with diode-pumped lasers (Coherent, Santa Clara, CA, USA) operating at 488 nm and 532 nm, respectively. Fluorescence images with two different excitation beams were sequentially collected with a 100X oil-immersion objective (NA 1.35). A dichroic beam splitter (Di01-R405/488/532/635, Semrock, Rochester, NY, USA), together with a notch filter (NF03-405/488/532/635, Semrock, Rochester, NY, USA), was used to remove the excitation beam. Images were recorded with an EMCCD (Andor Ixon, DU897ECS) for 0.2 s integration time in a given z section and taken automatically with z sections of every 300 nm across the cell depth (typical cell height was less than 10 μm, and 55 frames were collected) using a PI motion controller (E-709, C-867, Physik Instrumente, Karlsruhe, Germany). Total mRNA per cell was analyzed using FISH–quant software as reported previously [31]. Briefly, the outline of cells was defined, and the background of an image was estimated and subtracted by smoothing with a large Gaussian kernel (5X SD of point spread function). Then, the signal-to-noise ratio (SNR) of resulting images was further enhanced by filtering with a small Gaussian kernel (1X SD of Gaussian fit of point spread function). Spot candidates were identified using non-maximal suppression based on local maximum (nonMaxSupr function of Piotr’s Matlab toolbox). The size of the region around each detected spot was set to 400 nm in the lateral dimension and 600 nm in the axial dimension. The minimum intensity of a spot candidate was specified from the plot of the number of detected mRNAs versus the corresponding threshold value. The number of mRNAs less sensitive to threshold movement was chosen in the near plateau. A threshold was manually determined in individual cells so that the mRNA molecules detected with given thresholds could be well matched with the spots identified from raw fluorescent signals. The predetected spots were fitted with a 3D Gaussian function; then parameters including Gaussian width, position, amplitude, and local background, were restricted to a reasonable range to select appropriate spots. Finally, the minimum distances between spots were set to 300 nm in x, y, and z axes to prevent multiple counting of spots that could be in close proximity to each other.

### 2.9. SDS-PAGE and Western Blot Analysis

Treated cells were washed with ice-cold PBS (Welgene, Gyeongsan, Korea), scraped in 150 μL of RIPA buffer (Biosesang, Seongnam, Korea), and further incubated on ice for 30 min. Cell debris was removed by centrifugation at 15,000 rpm for 15 min. Equal amounts of total protein determined by BCA protein assay (Thermo Scientific, Waltham, MA, USA) were subjected to SDS-PAGE and transferred to polyvinylidene fluoride (PVDF) membranes. Each membrane was blocked with 5% nonfat dry milk in Tris-buffered saline containing 0.1% Tween-20 and incubated with primary antibodies overnight at 4 °C. Blots were developed with an HRP-conjugated secondary antibody, followed by visualization using enhanced chemiluminescence (ATTO KOREA, Daejeon, Korea).

### 2.10. Subcellular Fractionation

Cytoplasmic and nuclear fractions were collected using NE-PER^TM^ nuclear and cytoplasmic extraction reagents (Thermo Scientific, Waltham, MA, USA) as described in the manufacturer’s instructions. Briefly, cells were harvested using trypsin-EDTA and subsequently washed with chilled PBS. Cytosolic proteins were first extracted by disrupting cell membranes, followed by centrifugation. Intact nuclei were washed with cold PBS and lysed with high-salt NE-PER buffer. Individual fractions were confirmed by Western blotting with antibodies against vinculin (cytosolic extract) and LaminB1 (nuclear extract).

### 2.11. Quantification of Relative mRNA ratio of PKM Isoform by qRT-PCR

pET-28a-hPKM1 plasmid (#44241) was purchased from Addgene Inc. (Watertown, MA, USA). mCherry-PKM2 plasmid (pCMV-PKM2-mCherry) was prepared by inserting the PKM2 gene (Clone ID hMU009169, Korea human gene bank) to pCMV-mCherry using Xho1 and EcoR1 sites. Plasmids of PKM isoforms were quantified using a NanoDrop Lite spectrophotometer (Thermo Scientific, Waltham, MA, USA). Copy number was calculated using the following formula:

$$\mathrm{Number}\;\mathrm{of}\;\mathrm{copies}\;\left(\mathrm{molecules}\right)=\frac{X\;\mathrm{ng}\;\times \;6.0221\;\times \;10^{23}\;\mathrm{molecules}/\mathrm{mole}}{\left(N\;\times \;660\;g/\mathrm{mole}\right)\;\times \;1\;\times \;10^{9}\;\mathrm{ng}/g}$$

where **X** = amount of plasmid (ng), **N** = size of the plasmid (bp), and 660 g/mole corresponds to an average mass of 1 base pair (bp) dsDNA. Plasmids were serially diluted with nuclease-free water (Thermo Scientific, Waltham, MA, USA) from 1 × 10^9^ to 1 × 10^5^ copies to generate a standard curve. qRT-PCR was performed using a QuantStudio 1 system (Applied Biosystems, Waltham, MA, USA); the detailed reaction conditions were 95 ℃ for 2 min followed by 40 cycles of denaturation at 95 ℃ for 15 s and annealing at 57 ℃ for 60 s. For absolute quantification, the same amount of cDNA synthesized from HCT116 cell lysates was amplified as described above. The number of PKM mRNA isoforms in HCT116 cells was calculated from the linear regression of the standard curve by converting the Ct value to plasmid copy number.

### 2.12. Statistical Analysis

The RT-PCR and Western blotting results were quantified using ImageJ software. Band densitometric data are expressed as mean ± standard deviation of mean (SD) from at least three independent experiments. SM-FISH-STIC data are expressed as mean ± standard deviation of mean (SEM). Statistical significance between the groups in terms of expression level was verified by pairwise comparison using a Student’s t-test. *p*-values less than 0.05 were considered statistically significant.

## 3. Results and Discussion

### 3.1. Two Splicing Isoforms of PKM Can Be Distinctly Quantified at a Single-Cell Level

**Figure 1:**
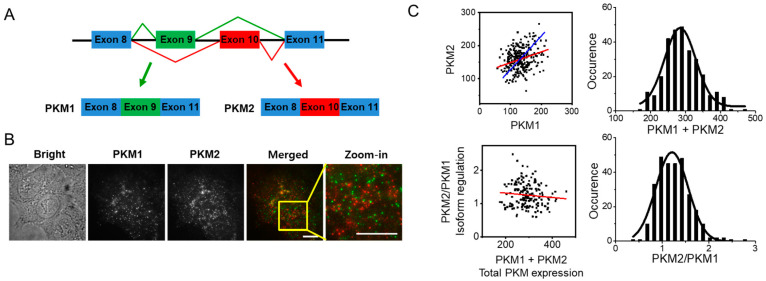
Detection of endogenous mRNAs of PKM isoforms in HCT116 cells using SM-FISH-STIC. (**A**) Schematic diagram of alternative splicing of PKM isoforms. PKM1 and PKM2 are produced via alternative splicing of the mutually exclusive exons 9 and 10. (**B**) Fluorescence images of single PKM mRNAs in HCT116 cells. PKM1 was detected with AX488 (green), and PKM2 was detected with Cy3. Images were processed as described in the Materials and Methods section and visualized using a maximum intensity projection of the entire *z*-axis of the cells (20 μm). Scale bar, 10 μm. (**C**) (**Left**) 2D plot of PKM isoforms in HCT116 cells. Each point corresponds to the number of mRNA molecules of each PKM isoform in individual cells. A linear regression of all the points is shown as a red line. The slope of the blue line corresponds to 1.2 (the average ratio of PKM2 to PKM1), and it passes through the average value of each PKM isoform (132, 159). (**Right**) Distribution of the total number of PKM isoform molecules (**top**) and the relative ratio of PKM2 to PKM1 (**bottom**) in individual cells.

Although single-molecule RNA imaging was developed to perform RNA sequencing at the single-cell level [25], quantification of splice isoforms remains challenging [32]. The detection and quantification of mRNA isoforms generated by alternative splicing at the single-cell level was first reported by Waks et al. [33]. In a previous study, two long isoforms (>1000 bp) that differed substantially in their RNA sequences (>800 base pairs (bp)) were selected for SM-FISH such that more than 50 sequential probes specific for each isoform were utilized for easy detection. The RNA sequences of the two PKM isoforms differ by only 22 amino acids (Figure 1A). Even within this region, most of the base pairs match, as denoted by the asterisks in Figure S1A. We chose the 20 bp sequence with the least overlap (highlighted in the red box in Figure S1A) as a target sequence for the two PKM isoforms. To detect such a short target sequence at the single-mRNA level, we utilized SM-FISH with sequential tethered and intertwined oligonucleotide complexes (SM-FISH-STIC; Figure S1B) [34]. In this method, the first probe binds to the target containing three repeats of a 35 bp sequence that is complementary to the secondary probe. Next, a secondary probe with five repeats of a distinct sequence for the fluorophore-coupled tertiary probe is added. In principle, 15 tertiary probes with fluorophores can be attached to a single target mRNA, providing a strong signal for single-molecule detection.

We first explored whether our probes bound specifically to the target mRNA and resulted in a strong signal for single-mRNA detection. As a model system, we chose HCT116 colorectal cancer cells for the following reasons. First, drug (GE) resistance in this cell line has been extensively studied [17,35,36]. In addition, the relative mRNA and protein levels of PKM1 and PKM2, as measured by quantitative PCR and Western blot, respectively, were comparable, which could help to minimize any experimental error in the quantification of the two isoforms (Figure S2). In the absence of either the primary or secondary probe, only 4–12 distinct spots (4–7% of the total number of molecules) were detected (Figure S3). However, PKM1 and PKM2 were simultaneously detected when all the probes were added sequentially (Figure 1B,C). We extended any fluorescent spots larger than the diffraction limit (~400 nm, or 4 pixels), which likely represent active transcription sites for both PKM isoforms in the nucleus. Based on the intensities of single Alexa Fluor488 (AX488) or Cy3 molecules (Figure S4), each RNA molecule was calculated to have approximately seven probes attached.

To confirm that the fluorescent spots that we observed with SM-FISH-STIC were specific to each PKM isoform, we used two additional sequence, targeting regions (exon 3–4 and 7) common to the two PKM isoforms (see Table S1 for detailed sequence information), and we designed probes for the simultaneously detection of both isoforms. In this scenario, when the isoform-specific PKM1 and pan-PKM probes are added simultaneously, the fluorescent spots detected by the PKM1 probe should colocalize with the fluorescent spots detected by the pan-PKM probe. Importantly, after optimizing the labeling protocol to reduce non-specific binding, we found that the two isoform-specific probes rarely overlapped (<10% of spots; Figure S5), suggesting that each probe selectively binds to the desired PKM isoform. Figure S5 shows that 82% of mRNA molecules that were labeled by the AX488-conjugated PKM1 probe were colocalized with the pan-PKM probe conjugated with Cy3. Given the non-specific binding discussed above (Figures S3 and S5), this error rate is comparable to that reported in previous studies [33]. We also confirmed that the intensity histograms of PKM1 and PKM2 were not affected by cell viability or cell status (drug treatment, Figure S4).

We further validated that the relative expression of the two PKM mRNA isoforms measured by SM-FISH-STIC was comparable to the value estimated by qRT-PCR. Full-length PKM1 and PKM2 mRNAs were prepared by in vitro transcription using the corresponding plasmids as templates. These mRNAs were serially diluted and used to create a set of standard curves for the quantification of their corresponding mRNAs in HCT116 cells (Figure S6A). The relative ratio of PKM2 to PKM1 mRNAs calculated by qRT-PCR was approximately 1.8 (Figure S6B). The average ratio calculated by SM-FISH-STIC, by counting each individual mRNA, was 1.2, which is comparable to the value obtained by qRT-PCR (Figure 1C). It has been reported that the mRNA and protein levels of PKM2 in colorectal cancer can be >100-fold higher than those of PKM1 [22,24,37]. When we measured the relative ratio of PKM2 to PKM1 mRNAs in two other colorectal cancer cell lines, HCT15 and Caco-2, the values were approximately 4 and 20, respectively (Figure S6C). These data suggest that the relative ratio of PKM2 to PKM1 mRNAs can be differ considerably depending on the cell line, even in the same tissue type.

Because we simultaneously quantified the PKM1 and PKM2 mRNA levels in each cell, we next investigated the cell-to-cell heterogeneity at the single-cell level. The number of mRNA molecules of each PKM isoform was counted and plotted in 2D (Figure 1C). The levels of PKM1 and PKM2 mRNA were heterogeneous at the single-cell level; the total number of PKM isoforms (PKM1 + PKM2) in the cell with the highest expression was approximately twofold higher than that of the cell with the lowest expression. Furthermore, the relative ratio of PKM2 to PKM1 varied from 0.5 to 2.8, meaning that the predominant PKM isoforms and their glycolytic activity also varies from cell to cell. When the PKM1 and PKM2 mRNA levels in all individual cells were plotted together, we observed a positive correlation (red line), suggesting that the PKM2/PKM1 ratio depends on the expression of PKM1. The PKM2/PKM1 ratio slightly decreased with a slope of −6.7 × 10^−4^ when total PKM expression increased (Figure 1C) As a reference, a blue line with a slope of 1.2 was also plotted, which was based on the average relative ratio of PKM2 to PKM1. Based on these measurements, we confirmed that the degree of PKM isoform expression, as well as that of the predominant isoform, was heterogeneous at the single-cell level and cannot be simply explained by the average value.

### 3.2. Depletion of PKM1 Induces a Heterogeneous PKM2 Increase

**Figure 2:**
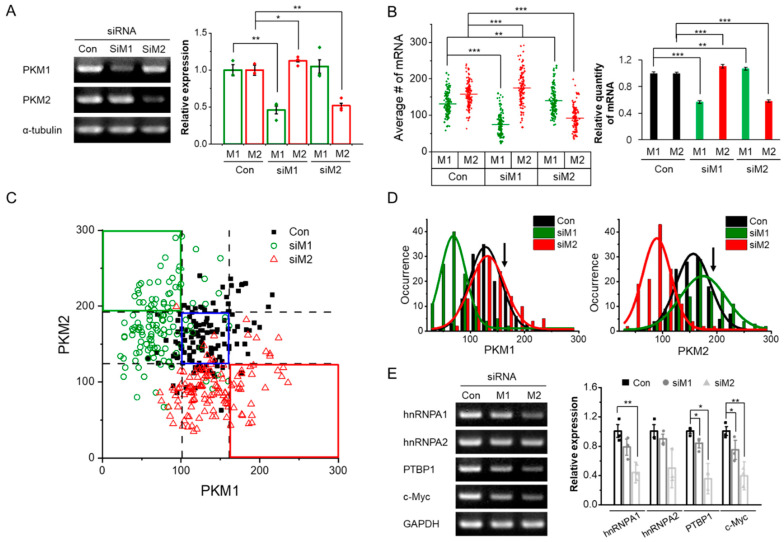
Effects of isoform-specific knockdown on PKM mRNA expression. Cells were treated with a scrambled siRNA (Con), PKM1-specific siRNA (SiM1), or PKM2-specific siRNA (siM2). (**A**) RT-PCR analysis of PKM isoforms. M1 and M2 corresponds to PKM1 and PKM2, respectively. Error bars represent average ± SD (*n* = 3) (**B**) Quantitative SM-FISH-STIC analysis of PKM isoforms. The number of mRNA molecules of each PKM isoform in individual cell was counted, and the average value is presented. M1 and M2 corresponds to PKM1 and PKM2, respectively. Error bars represent average ± standard error of the mean (SEM) (*n* ≥ 122) (**C**) 2D plot of each PKM isoform in HCT116 cells. Each point corresponds to the number of PKM isoforms in individual cell. The region enclosed within the dotted lines (the average ± SD in control cells) is highlighted in blue. The green box represents cells with a high expression level of PKM2 when PKM1 was depleted (greater than the average value + SD in control cells), and the red box represents cells with a high expression level of PKM1 when PKM2 was depleted (greater than the average value + SD in control cells). (**D**) Distribution of PKM1 (**left**) and PKM2 mRNA (**right**) from individual cells. The black, dark green, and red lines represent the Gaussian fits of each distribution. The black arrows indicate the corresponding average value + SD of the control. (**E**) Determination of relative mRNA levels of regulatory proteins involved in PKM splicing determined by RT-PCR. Data are shown as average ± SD of three independent experiments.* *p* < 0.05, ** *p* < 0.01, and *** *p* < 0.001.

Individual mRNAs of PKM isoforms can be quantified using SM-FISH-STIC, which allowed us to investigate how knockdown of one isoform by siRNA treatment affects the expression of the other isoform at the single-cell level. First, the knockdown efficiency was confirmed by RT-PCR (Figure 2A). Consistent with reported data with the same siRNAs [29], the mRNA levels of PKM1 and PKM2 were decreased to 46% and 52%, respectively, compared with the levels in control cells treated with scrambled siRNA. Interestingly, depletion of PKM2 did not affect the expression of PKM1 (siM2 in Figure 2A), but PKM1 depletion increased PKM2 expression (siM1 in Figure 2A). To examine the effects of PKM1/2 knockdown by SM-FISH-STIC, we treated cells with the specific siRNA for each isoform and counted the number of fluorescent spots for mRNA quantification. In control cells, the average numbers of PKM1 and PKM2 mRNA molecules were 132 ± 3 and 159 ± 3, respectively (Figure 2B). The total number of PKM1 and PKM2 mRNA molecules decreased by 57% and 58%, respectively, after siRNA treatment, comparably to the RT-PCR measurements. These data also support the finding that PKM isoforms can be quantitatively determined by SM-FISH-STIC.

To evaluate the heterogeneity of PKM1/2 expression, the number of mRNA molecules of each PKM isoform in individual cells was counted and plotted in 2D (Figure 2C). The vertical and horizontal dotted lines represent the average number of mRNA molecules of the two PKM isoforms ± the standard deviation (SD) in control cells. Interestingly, a small fraction of cells treated with either PKM1 (siM1) or PKM2 (siM2) siRNA had the same amount of PKM1 and PKM2 mRNA as the control cells (6% for PKM1 and 4% for PKM2; blue box in Figure 2C), suggesting that most cells were affected by siRNA treatment. Furthermore, we identified cells with enhanced expression of one isoform when the other isoform was depleted (green and red boxes in Figure 2C). For example, in cells treated with siM1, 28% of cells (green box in Figure 2C) had more than 192 molecules (the average + SD in control cells) of PKM2 mRNA. The distribution of PKM2 mRNA in siM1-treated cells was also wider than that in untreated cells, suggesting that siM1 treatment enhanced the heterogeneity of PKM2 expression; the mean value was also right-shifted (Figure 2D, right). However, when cells were treated with siM2, only 19% of the cells (red box in Figure 2C) had more than 161 molecules (the average + SD in control cells) of PKM1 mRNA, and the distribution of PKM1 in siM2-treated cells was comparable to that of control cells, although the width of the distribution increased slightly (Figure 2D, left). These data suggest that HCT116 cells are more sensitive to depletion of PKM1 mRNA than PKM2 mRNA and that PKM2 mRNA production increases to compensate for the loss of PKM1 mRNA, resulting in heterogeneous levels of PKM2 expression. One possible explanation for the enhanced heterogeneous PKM2 expression in PKM1 knockdown cells is that the ratio of PKM2 to PKM1, i.e., glycolytic activity, in HCT116 cells is quite low compared with other colorectal cell lines (Figure S6), which may imply that the high-activity pyruvate kinase PKM1 is required for their survival, making HCT116 cells more sensitive to depletion of PKM1 than depletion of PKM2. A recent study by Morita et al. demonstrated that PKM1, rather than PKM2, is required for glucose catabolism and cancer cell proliferation [38].

On the other hand, various studies have reported that depletion of one isoform affects the expression level of the other isoform. It has been reported that PKM2 knockout mice display compensatory and partially enhanced expression of PKM1 [39,40,41]. Zhou et al. reported that depletion of PKM1 mRNA in T24T cells caused increased expression of PKM2 mRNA [29]. In our case, the change in PKM1 mRNA levels in response to PKM2 knockdown was less pronounced, consistent with a previous report with respect to HCT116 cells [28]. In addition, HCT116 cells were more sensitive to depletion of PKM1, showing enhanced expression of PKM2 with increased cell-to-cell heterogeneity, consistent with the previous study [28]. To further examine whether increased PKM2 mRNA expression in response to knockdown of PKM1 is due to selective alternative splicing toward the PKM2 isoform, we measured the levels of hNRNPA1, hNRNPA2, PTBP1, and c-Myc, all of which have been reported to promote selective alternative splicing toward the PKM2 isoform [42,43]. Knockdown of PKM1 resulted in decreases in all of these proteins (Figure 2E), suggesting that the increase in PKM2 mRNA was not the result of enhanced alternative splicing toward the PKM2 isoform. Therefore, it is possible that other master regulators, such as specificity protein 1 (Sp1), hypoxia-inducible factor-1alpha (HIF-1α), and peroxisome proliferation-activated receptor gamma (PPARγ), rather than splicing-related proteins, are involved in the regulation of PKM mRNA expression (see [44] for review).

### 3.3. Population Shifts of PKM Isoforms Can Be Identified during Drug Resistance Acquisition

**Figure 3:**
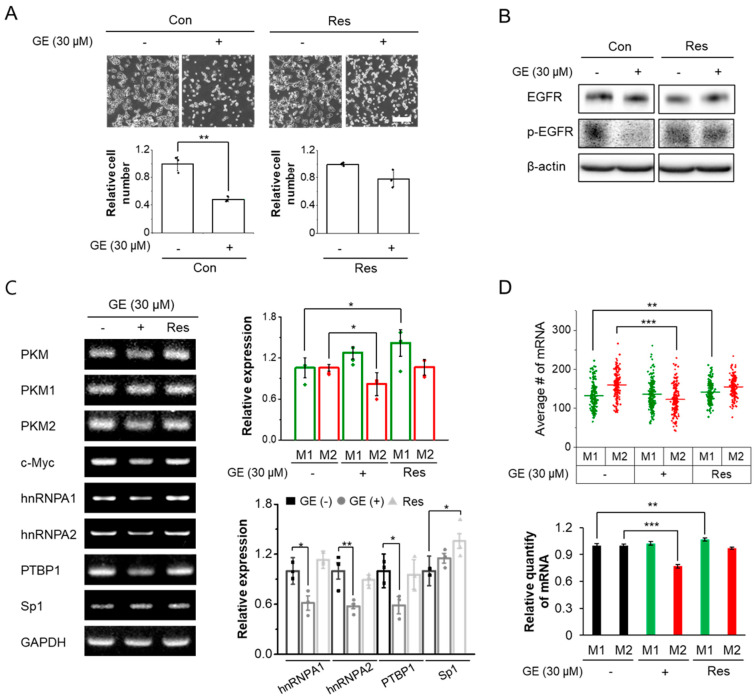
Effects of GE on the mRNA levels of PKM isoforms and proteins involved in PKM regulation. Cells were untreated (−) or GE-treated for 24 h (+). GE-resistant cells (GE Res) were also analyzed. (**A**) Evaluation of the cell viability. HCT116 cells (Con) or GE-resistant HCT116 cells (Res) were cultured in the absence or presence of 30 μM GE for 24 h, and viable cells were counted as described in the Materials and Methods section. Data are shown as average ± SD of three independent experiments. Scale bar, 200 μm. (**B**) The effect of GE on cell viability. HCT116 cells (Con) or GE-resistant HCT116 cells (Res) were treated with 30 μM GE for 24 h; then, the degree of EGFR expression and activation was measured by Western blotting. Untreated cells were used as a negative control. (**C**) RT-PCR analysis for PKM isoforms, c-Myc, hnRNPs, PTBP1, and Sp1. M1 and M2 in the graph correspond to PKM1 and PKM2, respectively. (**D**) Quantitative SM-FISH-STIC analysis of PKM isoforms. M1 and M2 correspond to PKM1 and PKM2, respectively. Error bars represent average ± SEM (*n* ≥ 162). * *p* < 0.05, ** *p* < 0.01, and *** *p* < 0.001.

Finally, we explored how anticancer drugs affect the alternative splicing of PKM isoforms. GE, a small molecule targeting the epidermal growth factor receptor (EGFR) signaling pathway [45], was chosen for the following reasons. First, among various tumors, EGFR is estimated to be overexpressed in 60–80% of colorectal cancers and is associated with a poor prognosis [46]; therefore, we hypothesized that HCT116 colorectal cancer cells with a high PKM1/PKM2 ratio might be ideal for our experiments. It should be noted that the K-RAS mutation (KRASp.G13D) of HCT116 does not influence the sensitivity to anti-EGFR treatment (especially with cetuximab), in contrast to the other K-RAS mutations [47]. Second, EGRF activation promotes PKM2 mRNA splicing via PKCε and NF-κB signaling in breast cancer, prostate cancer, and glioblastoma cells [48]. EGFR activation promotes PKM2 nuclear translocation, which results in increased transcription of c-Myc [49], a major regulator of PTB, hnRNPA1, and hnRNPA2, which are all involved in selective splicing toward the PKM2 isoform [42]. Furthermore, nuclear localization of PKM2 plays a critical role in GE resistance in colorectal cancer cells [17]. These previous studies imply that EGFR signaling can shift alternative splicing of PKM toward PKM2 expression. To further evaluate how the alternative splicing of PKM contributes to the acquisition of drug resistance, GE-resistant HCT116 cells (GE Res) were prepared, as described in the Materials and Methods section. HCT116 cells showed decreased EGFR phosphorylation, and only approximately 50% of cells survived after 24h of treatment with 30 μM GE, consistent with the IC50 measurement reported in a previous study [17] (Figure 3A). EGFR expression and phosphorylation were comparable in GE Res and wild-type HCT116 cells, but upon GE treatment, EGFR activation did not decrease in GE Res (Figure 3B).

We first examined the effect of GE using conventional RT-PCR. Upon GE treatment of HCT116 cells, mRNA levels of PKM2, as well as upregulators of PKM2 mRNA (hnRNPA1, hnRNPA2, PTBP1, and c-Myc) were all reduced, whereas PKM1 mRNA levels were rarely affected (statistically insignificant). As a result, the ratio of PKM2 to PKM1, i.e., glycolic activity, decreased (Figure 3C). These results are in agreement with previous studies reporting that alternative splicing toward PKM2 was reduced when expression of c-Myc and the above-mentioned splicing regulators decreased [42,49]. In the case of the GE Res, however, the expression of PKM2 and the splicing regulators recovered to the levels in control cells. In addition, PKM1 mRNA expression was further increased. Therefore, we speculated that transcription of the *PKM* gene increased [50], and the mRNA level of Sp1, a transcription factor for PKM, was increased in both GE-treated wild-type cells and GE Res (Figure 3C). Next, average number of PKM1 and PKM2 mRNA from individual cells was quantified using SM-FISH-STIC (Figure 3D). Consistent with the RT-PCR data, GE-treated HCT116 cells showed decreased PKM2 mRNA expression, but the PKM1 mRNA expression was comparable to that of control cells. In GE Res, the average number of PKM1 mRNA molecules was increased, and the number of PKM2 mRNA molecules recovered to the levels in untreated cells.

**Figure 4:**
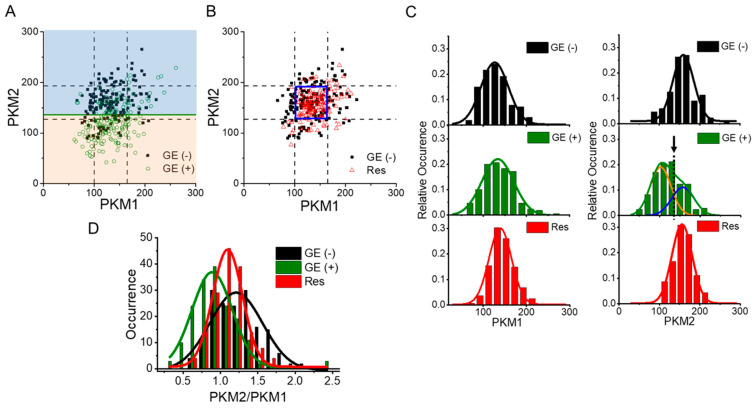
Effects of GE on the distribution of PKM isoforms. Cells were untreated (GE (−)) or GE-treated for 24 h (GE (+)). GE-resistant cells (GE Res) were also analyzed. (**A**) 2D plot of each PKM isoform in GE (−) or GE (+). Each point corresponds to the number of mRNA molecules of each PKM isoform in individual cells. The dotted lines represent the average value ± SD of each PKM isoform in GE (−). The dark green line represents the value at which the two PKM2 Gaussian fits (shown in orange and blue in Figure 4C) overlap. (**B**) 2D plot of each PKM isoform in GE (−) or GE Res. Each point corresponds to the number of mRNA molecules of each PKM isoform in individual cells. The dotted lines represent the average value ± SD of PKM isoforms from GE (−). (**C**) Distribution of PKM1 (left) and PKM2 mRNAs (right). The black, dark green, and red lines represent the Gaussian fit of each distribution. In the PKM2 mRNA distribution (right), the dark green line represents the sum of the two Gaussian fits (orange and blue lines) for GE (+). The black arrow highlights the overlap of the two Gaussian fits. (**D**) Distribution of the ratio of PKM2 to PKM1. The black, green, and red lines represent the Gaussian fits of each distribution.

When we examined the number of mRNA molecules of the PKM isoforms at the single-cell level, we found that two populations of GE-treated cells could be discriminated based on the distribution of PKM isoforms: a GE-sensitive population with decreased PKM2 (orange area in Figure 4A) and a GE-insensitive population (blue area in Figure 4A). The distribution of PKM isoforms in GE Res was also examined (Figure 4B). Overall, mRNA distributions of both PKMs were comparable in untreated cells and GE Res. Upon close examination, the populations of PKM1 in control cells and GE-treated cells were comparable but became narrower in GE Res and right-shifted compared with control cells (left in Figure 4C). However, GE-treated cells fell into two distinct populations based on the distribution of PKM2 mRNA (right side, Figure 4C). Furthermore, when we fit the distribution into the sum of two different Gaussian functions, one overlapped with the distribution of PKM2 mRNA in control cells. Approximately 41% of the GE-treated cells located in the blue area maintained their PKM2 mRNA level, and about 59% of GE-treated cells located in the orange area showed decreased PKM2 mRNA compared with pretreatment levels. On the other hand, the distribution of PKM2 mRNA in GE Res was narrower. Notably, the ratio of PKM2 to PKM1 mRNA, i.e., glycolytic activity, was decreased in GE Res compared with untreated cells (Figure 4D), although the levels of protein associated with PKM2 splicing (hnRNPA1, hnRNPA2, PTBP1, and c-Myc) were similar (Figure 3C). Control cells showed a broad PKM2/PKM1 distribution, but when cells were treated with GE, the PKM2/PKM1 distribution became narrower, and the ratio of PKM2 to PKM1 decreased, probably due to the decreased PKM2 in GE-sensitive cells. As the cells became resistant to GE treatment, the distribution narrowed further, suggesting that cells with tight regulation of PKM splicing and glycolysis can acquire GE resistance. In addition, enhanced PKM1 rather than PKM2 expression reduced glycolytic activity, which seemed to be beneficial for survival in the presence of GE.

**Figure 5:**
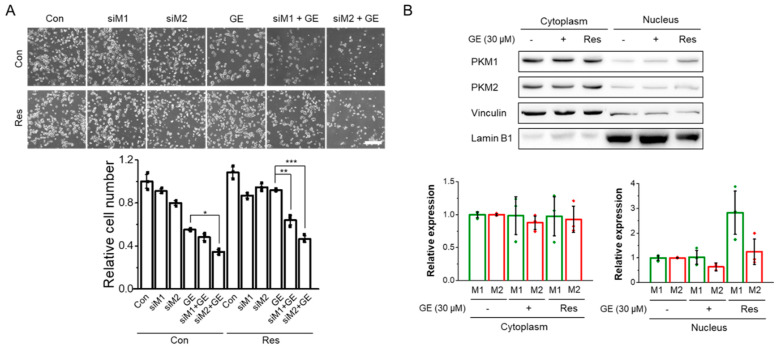
The importance of PKM isoforms in the acquisition of drug resistance. (**A**) The effect of PKM isoform depletion on the response to GE. Control or GE-resistant HCT116 cells (Con or GE Res) were pretreated with the appropriate siRNAs (siM1 and siM2) for 24 h and exposed to GE (30 μM) for 24 h. The viable cells were counted in three independent experiments as described in the Materials and Methods section. Data are shown as average ± SD of three independent experiments. Scale bar, 300 μm. (**B**) Expression of PKM proteins upon GE treatment in nuclear and cytoplasmic fractions. Individual fractions were normalized by vinculin (cytoplasmic extract) and Lamin B1 (nuclear extract). Data are shown as average ± SD of three independent experiments. * *p* < 0.05, ** *p*< 0.01, and *** *p* < 0.001.

To further elucidate the roles of PKM isoforms in drug resistance, we knocked down each isoform with siRNA and measured the viability upon GE treatment (Figure 5A). Knockdown of PKM1 did not affect the cell viability in response to GE treatment (statistically insignificant, fourth and fifth columns in Figure 5A). However, depletion of PKM2 caused a further decrease in cell viability, suggesting that PKM2 plays a role in survival upon GE treatment. On the other hand, once cells became resistant to GE (GE Res), both PKM1 and PKM2 were crucial to the maintenance of cell viability. Because previous studies suggest that nuclear localization of PKM2 plays a role in acquiring GE resistance [15,17,51], we also evaluated the cytoplasmic and nuclear localization of the PKM isoforms. Surprisingly, we found that nuclear PKM2 decreased with GE treatment, whereas PKM1 translocated to the nucleus in GE Res (Figure 5B). These data suggest that nuclear PKM1 might be involved in drug resistance.

Based on these data, we can infer the importance of PKM isoforms in the acquisition of drug resistance. First, we found that a GE-sensitive population of cells, located in the orange area in Figure 4A, was generated upon GE treatment, but once cells acquired GE resistance, this population disappeared (Figure 4B). Because PKM2 is critical for cell viability (Figure 5A), it is likely that the cells with decreased PKM2 expression (GE-sensitive) die during the continuous GE treatment protocol for production of GE Res. Given that GE inhibits EGFR activation, leading to reduced PKM2 transcription [52,53], GE sensitivity may correlate with sensitivity to EGFR inhibitors. In agreement with our data, previous studies reported the role of PKM2 in the acquisition of drug resistance. Pancreatic cancer cells with enhanced PKM2 expression were found to be resistant to gemcitabine-induced apoptosis [54]. Furthermore, depletion of PKM2 led to a reduction in cancer cell viability by increasing apoptosis [55], and maintaining appropriate PKM2 levels is critical for breast cancer cell survival [56].

On the other hand, it is likely that in GE-insensitive cells that are located in the blue area in Figure 4A, in which PKM2 expression is maintained, are selected and become the dominant cell type. Therefore, our data may support the hypothesis that pre-existing resistant subclones with high glycolytic activity are selected and expand after drug treatment [3]. In these cells, PKM1 expression is further increased through Sp1-dependent PKM transcription (Figure 3C), and the splicing of PKM isoforms is more tightly regulated, resulting in a narrower PKM2/PKM1 distribution (Figure 4D) with reduced glycolytic activity, exhibiting a more phenotypically homogenous population in GE Res. Furthermore, PKM1 that translocates to the nucleus (Figure 5B) may play a non-canonical role, such as acting as a transcriptional regulator, similarly to PKM2. Enhanced nuclear PKM1 is also observed in oxaliplatin-resistant DLD-1 colorectal cancer cells and other highly proliferative cell types, such as GepG2 and SMMC-7721 cells [20,57]. Interestingly, the nuclear localization of PKM1 is related to maintenance of stemness and differentiation potential in stem cells [58]. All of these data emphasize the role of PKM1 in the nucleus, and further studies are necessary to establish the role of nuclear PKM1 in drug resistance.

## 4. Conclusions

We established a method to quantify mRNAs of both PKM isoforms at the single-cell level and to reveal the role of each isoform and glycolytic activity in the acquisition of GE resistance in HCT116 cells. As expected based on their relatively low PKM2/PKM1 ratio compared with other colorectal cancer cells, knockdown of PKM1 caused a heterogeneous increase in PKM2 expression, suggesting that maintaining an appropriate level of PKM1 is more important than maintaining a consistent PKM2 level in HCT1116 cells. We identified two populations of HCT116 cells upon GE treatment: GE-sensitive cells with decreased PKM2 mRNA (decreased glycolytic activity) and GE-insensitive cells with stable levels of both PKM1 and PKM2 mRNA. Knockdown of PKM2 further sensitized HCT116 cells to GE treatment; therefore, PKM2 is crucial for survival in response to GE. However, GE Res showed enhanced PKM1 mRNA with a more homogenous PKM2/PKM1 distribution and PKM1 translocation to the nucleus, all of which imply that PKM1 with reduced glycolytic activity becomes dominant as HCT116 cells acquire GE resistance. Our approach reveals the importance of studying both PKM isoforms at the single-cell level, which reveals distinct subpopulations that cannot be observed by measurement of the entire cell population and highlights the roles of these isoforms during drug treatment. These data suggest that targeting both isoforms sequentially might inhibit the acquisition of GE resistance. Finally, this method could potentially be used to identify improved therapeutic molecules to treat drug-resistant cancer.

## Data Availability

Data can be provided from the corresponding author upon reasonable request.

## Supplementary Materials

The following supporting information can be downloaded at: https://www.mdpi.com/article/10.3390/biom12081082/s1, Figure S1: DNA sequences for PKM1 and PKM2 starting from 1141 bp to 1307 bp; Figure S2: mRNA and protein expression level of PKM isoforms in various cell lines; Figure S3: Specificity of the probe binding to each PKM isoform; Figure S4: Fluorescence intensity distributions of PKM mRNA; Figure S5: Colocalization analysis of PKM1 and PKM2; Figure S6: Quantification of PKM isoforms by qRT-PCR measurements; Table S1: SM-FISH probe sequences; Table S2: RT-PCR primer sequences. [42,59,60,61,62,63,64].
