# Supplementary material for: Single-Cell FISH Analysis Reveals Distinct Shifts in PKM Isoform Populations during Drug Resistance Acquisition
Source: Biomolecules. 2022 Aug 6;12(8):1082. doi: 10.3390/biom12081082 (PMC9405743; doi:10.3390/biom12081082)
Supplement: Supplementary file 1 [file biomolecules-12-01082-s001.zip › biomolecules-1825038-supplementary.pdf]

biomolecules

MDPI

Supplemental Information

Single-Cell FISH Analysis Reveals Distinct Shifts in PKM Isoform Populations during Drug Resistance Acquisition

Seong Ho Kim[1], Ji Hun Wi[1,2], HyeRan Gwak[1], Eun Gyeong Yang[1] and So Yeon Kim[1,3,*]

[1] Chemical and Biological Integrative Research Center, Biomedical Research Division, Korea Institute of Science and Technology, Hwarangno 14-gil 5, Seongbuk-gu, Seoul 02792, Korea
[2] Department of Biotechnology, College of Life Sciences and Biotechnology, Korea University, 145 Anam-ro, Seongbuk-gu, Seoul 02792, Korea
[3] Division of Bio-Medical Science and Technology, Korea University of Science and Technology (UST), KIST campus, Seoul 02792, Korea
* Correspondence: Correspondence: soyeonkim@kist.re.kr

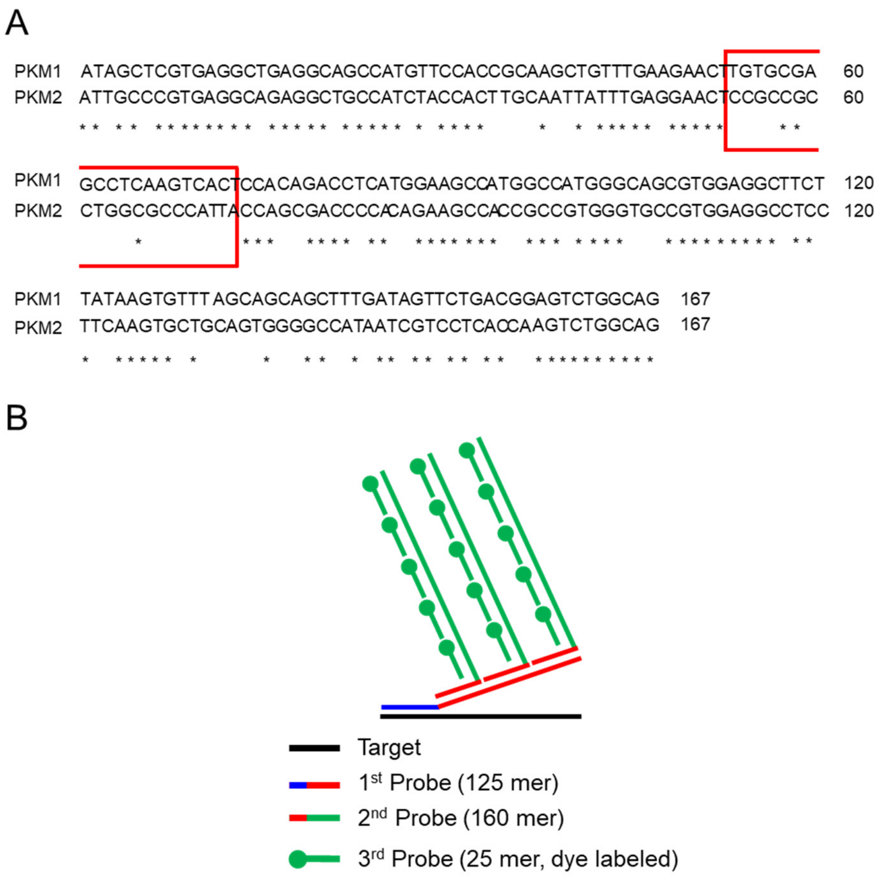

Figure S1. (A) DNA sequences for PKM1 and PKM2 starting from 1141 bp to 1307 bp, which corresponds to exon 9 and exon 10 region, respectively. Target-M1 and Target-M2 (see Table S1) for each isoform are highlighted in the red box. * denotes the overlapped sequence in PKM1 and PKM2. (B) Schematic presentation of probe binding to the target. .

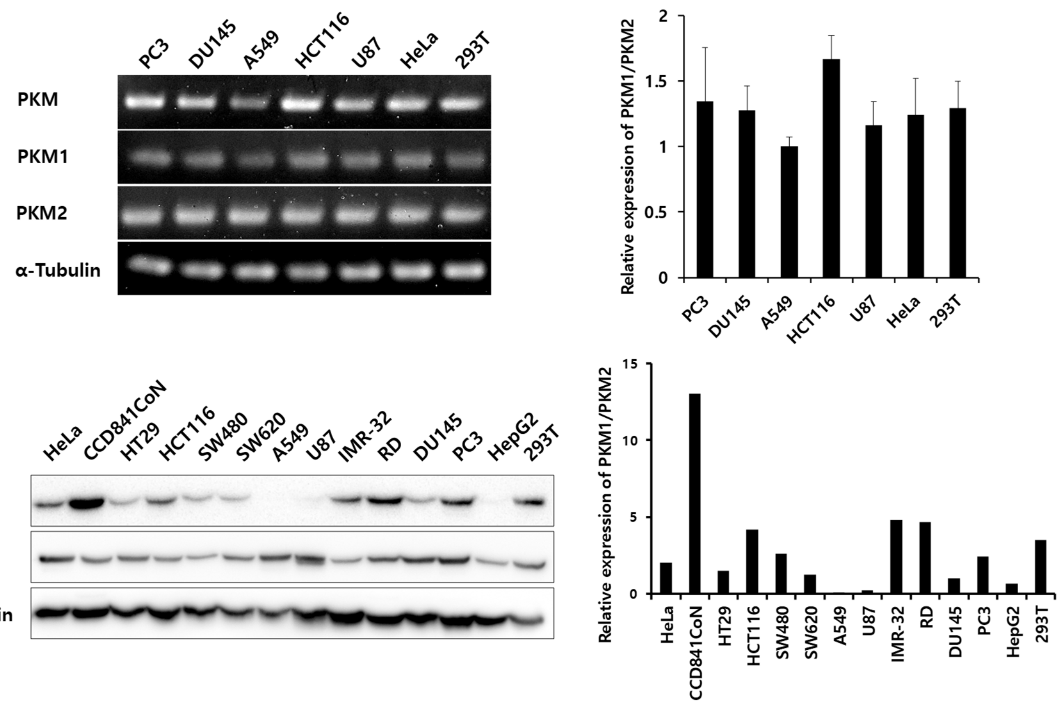

Figure S2. The mRNA and protein expression level of PKM isoforms in various cell lines.

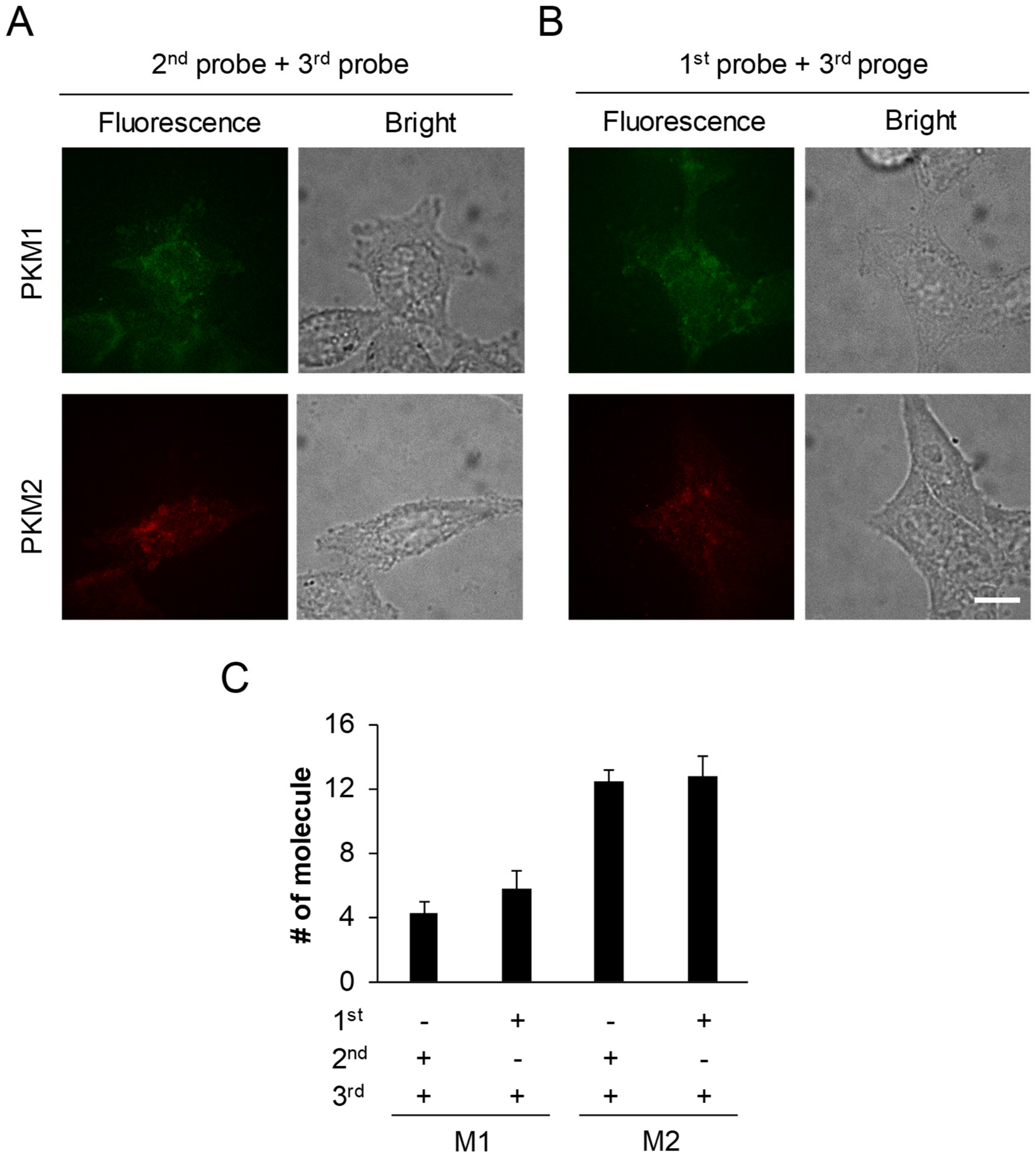

Figure S3. Specificity of the probe binding to each PKM isoform. (A) Primary probes for each PKM isoform were omitted, or (B) Secondary probes for each PKM isoform were omitted, but all the other components were sequentially added. The fluorescence images were taken as described in the Materials and Methods. (C) Quantification of the fluorescent spots from (A) and (B). M1 and M2 correspond to PKM1 and PKM2, respectively. Data are shown as average ± SEM. Scale bar, 10 μm.

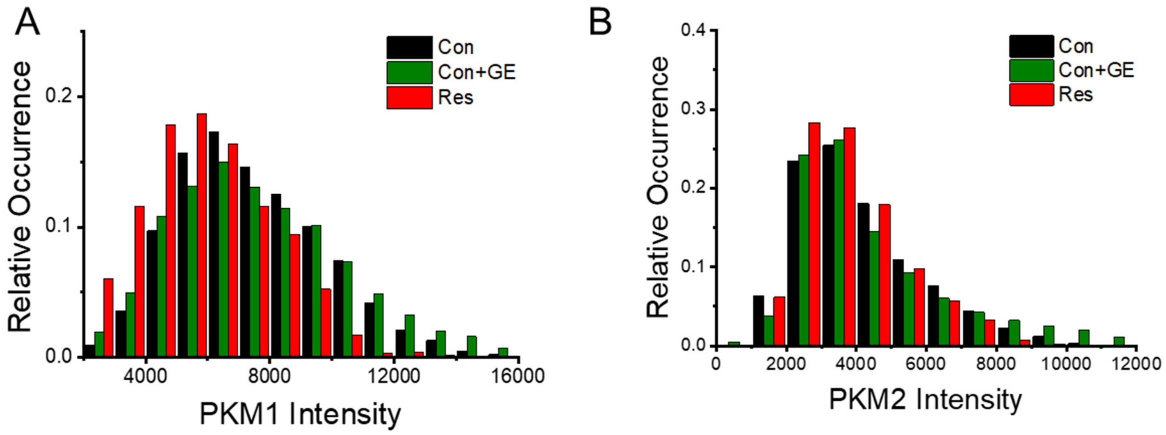

Figure S4. The fluorescence intensity distributions of PKM mRNA. The fluorescence intensity distributions of (A) PKM1 mRNA (probe: AX488) and (B) PKM2 mRNA (probe: Cy3) in HCT116 cells in different status (GE(-), GE(+), and Res).

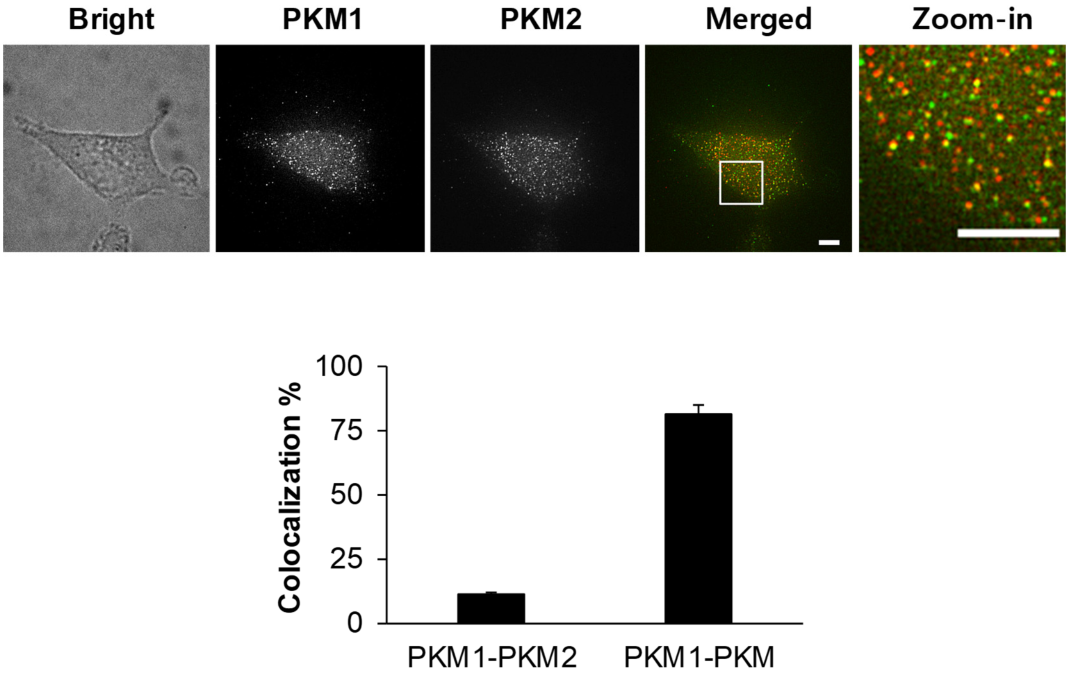

Figure S5. Colocalization analysis of PKM1 and PKM2. PKM1 mRNA was labeled with AX488, while PKM2 was labeled with Cy3. Colocalization was calculated by using ImagePro software (Media Cybernetics, USA). Data are shown as average ± SEM. Scale bar, 5 μm. .

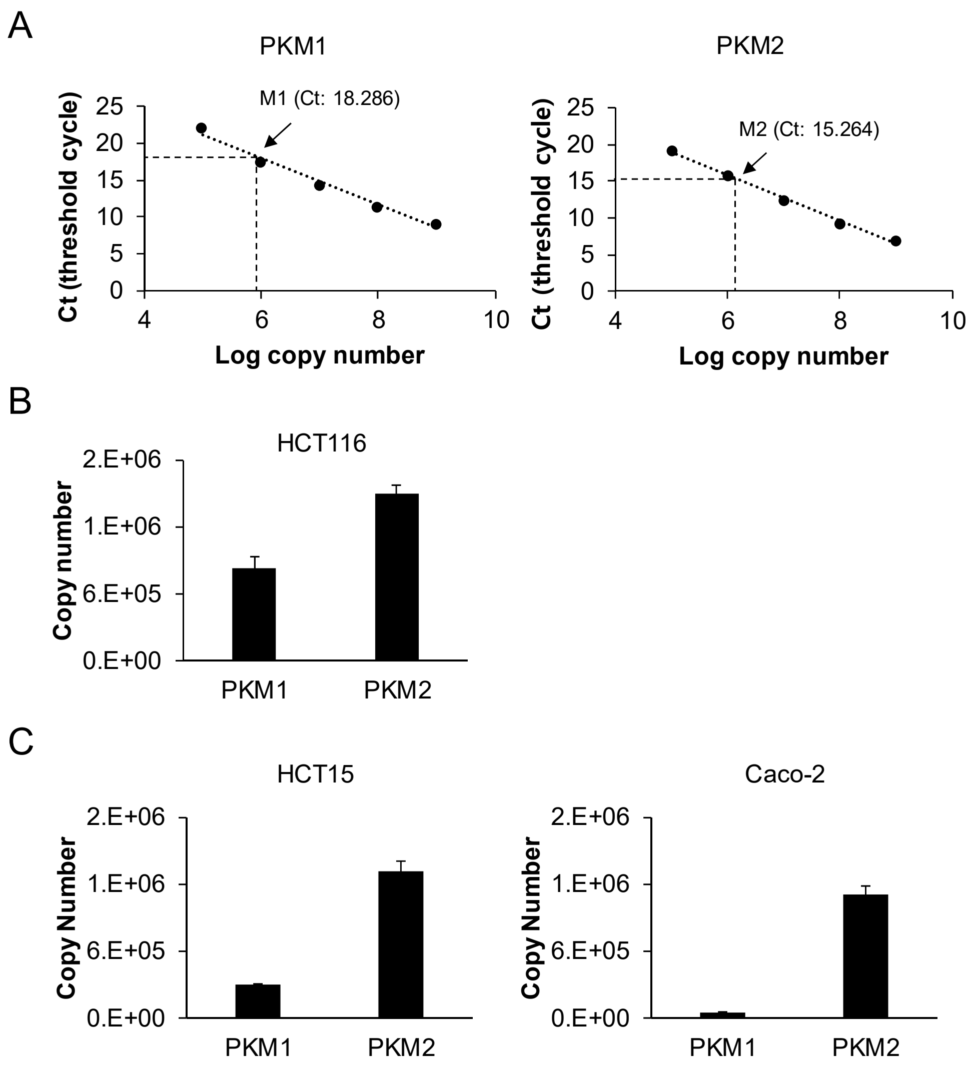

Figure S6. Quantification of PKM isoforms by qRT-PCR measurements. (A) Standard curves for each PKM isoform were obtained using a serial dilution of full-length plasmid DNA as templates. The average Ct values for each PKM isoform isolated from HCT116 cells were presented as black arrows. (B) Calculated copy numbers of each PKM isoform in HCT116 cells. (C) Calculated copy numbers of each PKM isoform in other colorectal cancer cell lines, HCT15 and Caco-2. Data are shown as average ± SD of three independent experiments.

Table S1. SM-FISH probe sequences. The sequences highlighted in blue, magenta, and light blue represent the target binding site for PKM1, PKM2, and pan-PKM, respectively. Other colors used in the sequence match with the colors shown in Figure S1.

**Nam**	**Sequence**
Target-M1	5'-**TGT GCG AGC CTC AAG TCA CT**
1st -M1	5'-**AGT GAC TTG AGG CTC GCA CA**T CGT TGG CCC CCG ACC GTT ACA GAC TGT TCT CAG TTC GTT GGC CCC CGA CCG TTA CAG ACT GTT CTC AGT TCG TTG GCC CCC GAC CGT TAC AGA CTG TTC TCA GT
2nd -M1	5'-ACT GAG AAC AGT CTG TAA CGG TCG GGG GCC AAC GAA CGC GAT TGA CTA CCA GAC TAT ACG ACG CGA TTG ACT ACC AGA CTA TAC GAC GCG ATT GAC TAC CAG ACT ATA CGA CGC GAT TGA CTA CCA GAC TAT ACG ACG CGA TTG ACT ACC AGA CTA TAC G
3rd -M1	5' -**Alexa488(AX488)**-CGT ATA GTC TGG TAG TCA ATC GCG T
Target-M2	5'-**CCG CCG CCT GGC GCC CAT TA**
1st -M2	5'-**TAA TGG GCG CCA GGC GGC GG**T CTA TAA ACG AGC AAT TAC ATA AGA CAT CCG TAG ATC TAT AAA CGA GCA ATT ACA TAA GAC ATC CGT AGA TCT ATA AAC GAG CAA TTA CAT AAG ACA TCC GTA GA
2nd -M2	5'-TCT ACG GAT GTC TTA TGT AAT TGC TCG TTT ATA GAT ACC AAT TCT GAC ATA TGT GAC TCA TAC CAA TTC TGA CAT ATG TGA CTC ATA CCA ATT CTG ACA TAT GTG ACT CAT ACC AAT TCT GAC ATA TGT GAC TCA TAC CAA TTC TGA CAT ATG TGA CTC A
3rd -M2	5'-**Cy3**-TGA GTC ACA TAT GTC AGA ATT GGT A
Target-panM-1	5'-**CTT CTC TCA TGG AAC TCA TGA GTA CCA TGC GGA GA**
1st-panM-1	5'-**TCT CCG CAT GGT ACT CAT GAG TTC CAT GAG AGA AGT** CTA TAA ACG AGC AAT TAC ATA AGA CAT CCG TAG ATC TAT AAA CGA GCA ATT ACA TAA GAC ATC CGT AGA TCT ATA AAC GAG CAA TTA CAT AAG ACA TCC GTA GA
Target-panM-2	5'-**TCA TGG TGG CTC GTG GTG ATC TAG GCA TTG AGA TT**
1st-panM-2	5'-**AAT CTC AAT GCC TAG ATC ACC ACG AGC CAC CAT GAT** CTA TAA ACG AGC AAT TAC ATA AGA CAT CCG TAG ATC TAT AAA CGA GCA ATT ACA TAA GAC ATC CGT AGA TCT ATA AAC GAG CAA TTA CAT AAG ACA TCC GTA GA

Table S2. RT-PCR primer sequences.

Name	Sequence
PKM1[1]	F:5′- GAGGCAGCCATGTTCCAC -3′<br>R:5′-TGCCAGACTCCGTCAGAACT-3′
PKM2[1]	F:5′-GGGTTCGGAGGTTTGATG-3′<br>R:5′-ACGGCGGTGGCTTCTGT-3′
PKM[2]	F: 5′-CTGAAGGCAGTGATGTGGCC-3′<br>R: 5′-ACCCGGAGGTCCACGTCCTC-3′
Sp1[3]	F: 5’- GCCTCCAGACCATTAACCTCAG-3’<br>R: 5’- TCATGTATTCCATCACCACCAG-3
hnRNPA1[4]	F: 5’- ACATATGCCACTGTGGAGGAG-3’<br>R: 5’- CTTGCTTTGACAGGGCTTTTC-3’
hnRNPA2	F: 5’-AAACGTGCTGTAGCAAGAGAG-3’<br>R: 5’-TGGTCCTGGTCCGAAATTTCC-3’
PTBP1	F: 5’-TTTGGGAAGGTCACCAACCTC-3’<br>R: 5’-CTGTGCCGAACTTGGAGAAAA-3
c-Myc[5]	F: 5’- CTGCCAAGAGGGCTAAGTTG-3’<br>R: 5’- AGCTTTTGCTCCTCTGCTTG-3’
α-tubulin[6]	F: 5’- AGCGTGCCTTTGTTCACT-3’<br>R: 5’- CACACCAACCTCCTCATAATCC-3
GAPDH[7]	F: 5′-CTGCACCACCAACTGCTTAGC-3′<br>R: 5′-CTTCACCACCTTCTTGATGTC-3′

References

1. Chaneton, B.; Hillmann, P.; Zheng, L.; Martin, A.C.L.; Maddocks, O.D.K.; Chokkathukalam, A.; Coyle, J.E.; Jankevics, A.; Holding, F.P.; Vousden, K.H.; et al. Serine is a natural ligand and allosteric activator of pyruvate kinase M2. *Nature* **2012**, *491*, 458-462, doi:10.1038/nature11540.
2. David, C.J.; Chen, M.; Assanah, M.; Canoll, P.; Manley, J.L. HnRNP proteins controlled by c-Myc deregulate pyruvate kinase mRNA splicing in cancer. *Nature* **2010**, *463*, 364-368, doi:10.1038/nature08697.
3. Zhao, W.-F.; Wang, H.-B.; Xie, B.; Hu, L.-J.; Xu, L.-H.; Kuang, B.-H.; Li, M.-Z.; Zhang, X. Sp1 and Sp3 are involved in the full transcriptional activity of centromere protein H in human nasopharyngeal carcinoma cells. **2012**, *279*, 2714-2726, doi:10.1111/j.1742-4658.2012.08654.x.
4. Loh, T.; Moon, H.; Cho, S.; Jang, H.; Liu, Y.; Tai, H.; Jung, D.-W.; Williams, D.; Kim, H.-R.; Shin, M.-G.; et al. CD44 alternative splicing and hnRNP A1 expression are associated with the metastasis of breast cancer. **2015**, doi:10.3892/or.2015.4110.
5. Kim, S.J.; Koo, O.J.; Park, H.J.; Moon, J.H.; Da Torre, B.R.; Javaregowda, P.K.; Kang, J.T.; Park, S.J.; Saadeldin, I.M.; Choi, J.Y.; et al. Oct4 overexpression facilitates proliferation of porcine fibroblasts and development of cloned embryos. **2015**, *23*, 704-711, doi:10.1017/s0967199414000355.
6. Kim, H.; Woo, D.J.; Kim, S.Y.; Yang, E.G. p21-activated kinase 4 regulates HIF-1alpha translation in cancer cells. *Biochem Biophys Res Commun* **2017**, *486*, 270-276, doi:10.1016/j.bbrc.2017.03.024.
7. Lee, J.-W.; Ryu, H.-C.; Ng, Y.C.; Kim, C.; Wei, J.-D.; Sabaratnam, V.; Kim, J.-H. 12(S)-Hydroxyheptadeca-5Z,8E,10E-trienoic acid suppresses UV-induced IL-6 synthesis in keratinocytes, exerting an anti-inflammatory activity. *Experimental & Molecular Medicine* **2012**, *44*, 378, doi:10.3858/emm.2012.44.6.043.
